# Draft Whole-Genome Sequence of Psychrotrophic *Arthrobacter* sp. Strain 7749, Isolated from Antarctic Marine Sediments with Applications in Enantioselective Alcohol Oxidation

**DOI:** 10.1128/genomeA.01197-17

**Published:** 2017-10-26

**Authors:** Diego E. Sastre, Leidaiany P. Santos, Edna Kagohara, Leandro H. Andrade

**Affiliations:** a Institute of Chemistry, Biochemistry Department, University of São Paulo, São Paulo, Brazil; b Institute of Chemistry, Chemistry Department, University of São Paulo, São Paulo, Brazil

## Abstract

Here, we report the 4.12-Mb draft genome sequence of *Arthrobacter* sp. strain 7749, isolated from marine sediment samples of the Antarctic Peninsula, using enriched medium with (*RS*)-1-(4-phenyl)-ethanol as a carbon source. This genome sequence will provide relevant information for applications in enantioselective alcohol oxidation to improve industrial catalytic processes.

## GENOME ANNOUNCEMENT

Actinobacteria belonging to the genus *Arthrobacter* constitute a group of Gram-positive, chemoorganotrophic, and cosmopolitan soil microorganisms exhibiting nutritional versatility, as demonstrated by their capacity to metabolize numerous substrates, including several toxic compounds, as carbon sources (1–3). *Arthrobacter* sp. strain 7749 was isolated from Antarctic marine sediments based on its ability to oxidize (*RS*)-1-phenylethanol derivatives with extremely high selectivity for (*S*)-enantiomers at low temperatures (4, 5). Thus, the genome report of the psychrotrophic strain 7749 will provide valuable information for the identification of cold-active and enantioselective oxidoreductases.

Genomic DNA of strain 7749 was extracted from a culture grown for 24 h at 20°C in Luria-Bertani (LB) broth using a Qiagen DNeasy blood and tissue kit (catalog number 69504), according to the manufacturer’s protocol for Gram-positive bacteria. The quantity, purity, and integrity of the genomic DNA were determined using a NanoDrop microvolume spectrophotometer (Thermo Fisher Scientific) and agarose gel ethidium-bromide stain. High-quality genomic DNA was used to construct a library with an approximate insert size of 500 bp using a Nextera XT DNA library preparation kit, according to the manufacturer’s protocol. The generated library was loaded onto the Illumina MiSeq sequencing platform, producing a total of 1,005,914 single reads of 250 bp, representing 61× coverage of the strain 7749 genome. The trimmed reads were assembled *de novo* into 16 large contigs using SPAdes 3.0.0 (6) implemented on the Orione Galaxy server (7) (k-mer values of 21, 33, 55, 77, 99, and 127), giving 4,125,942 bp total length and an *N*_50_ contig size of 525,170 bp, with a largest contig of 779,368 bp. The contigs were scaffolded using SPAdes 3.0.0 (6) and SSPACE version 3.0 (8) and were anchored onto the nearest reference genome of *Arthorobacter* sp. strain YC-RL1 (9) using CONTIGuator (10).

Based on phylogenetic sequence analysis of 16S rRNA and the housekeeping genes *recA*, *gyrB*, *rpoB*, *rpoD*, and *atpD*, strain 7749 was most closely related to *Arthrobacter* sp. YC-RL1 (9) and *Arthrobacter* sp. LS16 (11) strains.

The genome of strain 7749 consists of a single circular chromosome, with a G+C content of 60.7%. Functional annotation was performed using the Rapid Annotation using Subsystems Technology (RAST) server (12) and the NCBI Prokaryotic Genome Annotation Pipeline (13). A total of 3,616 candidate protein-coding sequences (CDSs) were predicted, and 62 RNA genes were identified, which are in accordance with other reported *Arthrobacter* sp. genomes.

Genomic analysis of *Arthrobacter* sp. strain 7749 revealed numerous genes putatively involved in the oxidation and reduction of organic compounds with useful characteristics for industrial applications, including 9 genes that were annotated as alcohol dehydrogenases, and more than 50 genes annotated as oxidoreductases.

In conclusion, this initial genome analysis of strain 7749 contributes to enrichment of the knowledge of the cold-active oxidoreductases performing enantioselective alcohol oxidation with potential application in agrochemical and pharmaceutical industries.

### Accession number(s).

The genome-sequencing project of *Arthrobacter* sp. strain 7749 has been deposited in GenBank under the accession number CP022462.
